# EVIDENCE-BASED REVIEWS & RECOMMENDATIONS FROM THE BRAZILIAN SOCIETY OF SHOULDER AND ELBOW SURGERY: LABRAL REPAIR FOR ANTERIOR SHOULDER INSTABILITY — MORE THAN TWO ANCHORS VERSUS UP TO TWO ANCHORS

**DOI:** 10.1590/1413-785220263401e308949

**Published:** 2026-08-31

**Authors:** Paulo Henrique Schmidt Lara, Paulo Santoro Belangero, Márcio Schiefer, Leonardo Zanesco, Marcos Rassi Fernandes, Joel Murachovsky, João Felipe de Medeiros, Eduardo Angeli Malavolta

**Affiliations:** 1 Escola Paulista de Medicina, UNIFESP, Sao Paulo, SP, Brazil.; 2 Instituto Nacional de Traumatologia e Ortopedia Jamil Haddad (INTO), Rio de Janeiro, RJ, Brazil.; 3 Universidade de Sao Paulo, Faculdade de Medicina, Hospital das Clinicas HCFMUSP, Sao Paulo, SP, Brazil.; 4 Universidade Federal de Goiás, Faculdade de Medicina, Goiânia, GO, Brazil.; 5 Faculdade de Medicina do ABC, Santo André, SP, Brazil.; 6 Universidade Federal do Rio Grande do Norte, Natal, RN, Brazil.

**Keywords:** Joint Instability, Shoulder Dislocation, Bankart Lesions, Suture Anchors, Arthroscopy, Instabilidade Articular, Luxação do Ombro, Lesões de Bankart, Âncoras de Sutura, Artroscopia

## Abstract

**Introduction:**

Anterior shoulder instability is a common condition that mainly affects young adults. It is most often traumatic and is frequently associated with a Bankart lesion, characterized by detachment of the anteroinferior glenoid labrum. When conservative treatment fails, surgical repair is usually indicated. Biomechanical studies suggest that a greater number of anchors may increase the contact area and provide greater stability between the labrum and the glenoid, promoting a more stable reconstruction of the capsulolabral complex.

**Methods:**

A systematic review was conducted in PubMed, Embase, Cochrane Library, and the Virtual Health Library (VHL) up to March 2026 to evaluate whether the use of more than two anchors is more effective than the use of up to two anchors for the arthroscopic treatment of anterior shoulder instability in adults. The certainty of the evidence was assessed with the GRADE approach.

**Results:**

One randomized controlled trial and two cohort studies were included. For function and complications, there was no evidence showing that the use of three or more anchors was more effective than the use of two anchors. For recurrence, there was evidence, although the certainty of the evidence was very low, that the use of three or more anchors was more effective than the use of up to two anchors.

**Conclusion:**

The surgeon should individually assess patient- and disease-related characteristics to make the best treatment decision. Regarding recurrence, the SBCOC Consensus Project suggests a preference for three or more anchors, given a trend toward a lower recurrence rate. ***Level of evidence II; Systematic review.***

## RESUMO

**Introdução:**

A instabilidade anterior do ombro é uma condição comum, que acomete principalmente adultos jovens. Na maioria das vezes, é de causa traumática e está frequentemente associada à lesão de Bankart, caracterizada pelo descolamento do lábio glenoidal anteroinferior. Quando o tratamento conservador falha, o tratamento cirúrgico é geralmente indicado. Estudos biomecânicos sugerem que um maior número de âncoras pode aumentar a área de contato e oferecer maior estabilidade entre o lábio e a glenoide, promovendo uma reconstrução mais estável do complexo capsulolabial.

**Métodos:**

Realizou-se revisão sistemática da literatura nas bases de dados PubMed, Embase, Cochrane Library e Biblioteca Virtual em Saúde (BVS) até março de 2026, com o objetivo de avaliar se a utilização de mais de duas âncoras é mais efetiva do que a utilização de até duas âncoras para o tratamento artroscópico da instabilidade anterior do ombro em adultos. A certeza da evidência foi avaliada pela abordagem GRADE.

**Resultados:**

Foram incluídos um ensaio clínico randomizado e dois estudos de coorte. Em relação aos desfechos de função e complicações, não foram encontradas evidências de que a utilização de três ou mais âncoras seja mais efetiva do que a utilização de duas âncoras. Em relação ao desfecho recidiva, existe evidência, ainda que de muito baixa qualidade, de que a utilização de três ou mais âncoras é mais efetiva do que a utilização de até duas âncoras.

**Conclusão:**

O cirurgião deve avaliar, de maneira individualizada, as características do paciente e da enfermidade para a tomada da melhor decisão de tratamento. Em relação ao desfecho recidiva, o Projeto Consensos SBCOC sugere preferência pela utilização de três ou mais âncoras, pois há tendência a uma menor taxa de recidiva. ***Nível de evidência II; Revisão sistemática.***

## INTRODUCTION

Anterior shoulder instability is a common condition that mainly affects young adults. It is most often traumatic and is frequently associated with a Bankart lesion, characterized by detachment of the anteroinferior glenoid labrum. When conservative treatment fails, surgical treatment is usually indicated. Currently, the most commonly used procedure is arthroscopic Bankart repair, which provides good clinical and functional results and lower morbidity when compared with open techniques.^1,2^

Arthroscopic repair of the glenoid labrum is based on reinsertion of the capsulolabral complex onto the glenoid rim using suture anchors. Several technical factors may influence the results of the procedure, including the position of the anchors, the tensioning of the joint capsule, the number of fixation points, and the number of anchors used. Among these, the number of anchors is considered potentially relevant because, in theory, a greater number of fixation points would provide better anatomical restoration and greater stability of the capsulolabral complex.^2–4^

Biomechanical studies suggest that a greater number of anchors can increase the contact area and offer greater stability between the labrum and the glenoid, promoting a more stable reconstruction of the glenoid labrum. For this reason, many surgeons have adopted the routine use of three or more anchors during arthroscopic repair. However, this approach may also increase the cost of the procedure, the operative time, and the potential risk of complications related to anchor placement, such as tunnel confluence or glenoid bone compromise.^3–5^

Despite these theoretical considerations, the available scientific evidence remains limited and shows inconsistent and conflicting results. Some clinical studies suggest that the use of three or more anchors may be associated with a lower rate of instability recurrence, whereas others demonstrate no significant difference in clinical outcomes compared with the use of two anchors. Moreover, most studies on the subject are observational, with relatively small sample sizes, which hinders a definitive interpretation of the results.^4–6^

Given these uncertainties, a critical synthesis of the available evidence on the impact of the number of anchors on the outcomes of arthroscopic repair for anterior shoulder instability is warranted. Therefore, the objective of this consensus is to evaluate whether the use of more than two anchors in labral repair is associated with better clinical outcomes and a lower recurrence rate when compared with the use of up to two anchors in adults undergoing arthroscopic repair of Bankart lesions.^2,4–7^

This study was conducted at the initiative of the Brazilian Society of Shoulder and Elbow Surgery with the participation of Cochrane Brazil.

### Research Question

Is the use of more than two anchors more effective than the use of two anchors for the arthroscopic treatment of anterior shoulder instability in adults?

## METHODS

- Databases searched: PubMed, Embase, Cochrane Library, and the Virtual Health Library (VHL).
- Descriptors: As described in Appendix 1.
- Search period: Until March 2026.
- Study selection: Updated systematic reviews (SRs) with adequate methodological quality were prioritized. In the absence of SRs, randomized controlled trials (RCTs) were considered, followed by observational studies.

## RESULTS

The search retrieved 604 references (279 in PubMed, 262 in Embase, 53 in the Cochrane Library, and 10 in the Regional Portal BVS).

**Figure 1:**
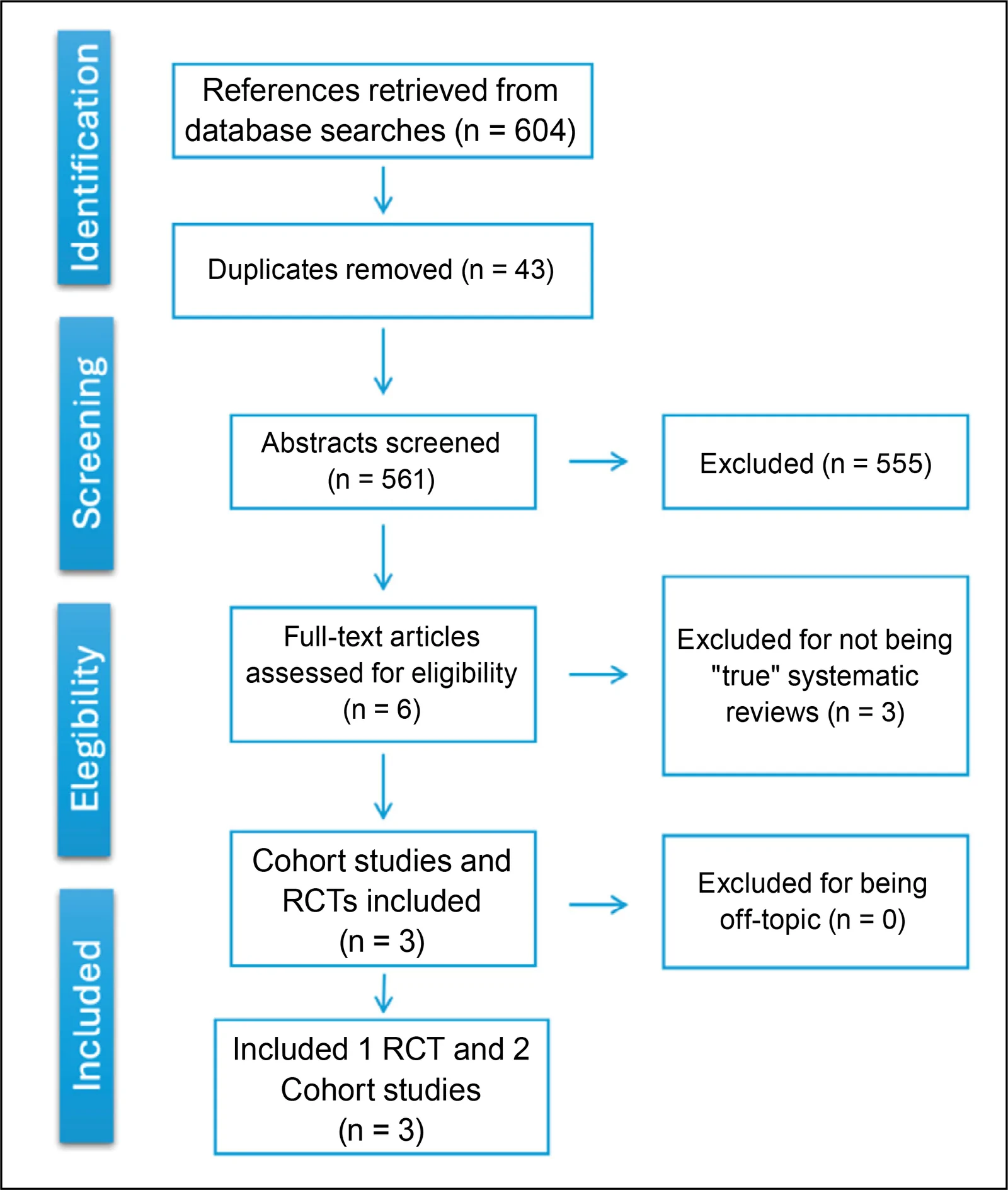
Flow diagram of the literature search.

After an initial assessment, 43 duplicate records were removed. Following abstract screening, 555 studies were excluded, leaving six studies for full-text evaluation. Three systematic reviews on the topic were assessed but not included, as they did not provide a direct comparison through randomized or comparative cohort data.^8–10^ One randomized controlled trial and two cohort studies were ultimately included (Figure 1).

## INCLUDED STUDIES

Buckup et al.^5^, in a randomized controlled trial, evaluated whether the use of three anchors would result in better anatomical reconstruction and better clinical outcomes than the use of two anchors in arthroscopic Bankart repair in patients with anterior shoulder instability. Two groups of 20 participants each were randomized, with a minimum follow-up of 12 months by magnetic resonance imaging (MRI) and 24 months by clinical evaluation. Group 1 received two double-loaded knotless anchors (3.5 mm), and Group 2 received three single-loaded knotless anchors (2.9 mm). On MRI, the three-anchor group showed anterior glenoid reconstruction closer to normal anatomy than the two-anchor group. For shoulder function, pain, range of motion, and functional scores, both groups achieved excellent clinical results, with no significant difference between them. Regarding recurrence, there was no statistical difference between the groups (two-anchor group: 2 recurrences; three-anchor group: 1 recurrence).

The authors concluded that both techniques provide good clinical results and that three anchors restore labral anatomy more closely, although this difference did not translate into a significant clinical benefit at two-year follow-up. The main reported limitations were the small sample size, single-center design, relatively short follow-up, and a technical difference between the implants used (anchor size).^5^ Vermeulen et al.,^11^ in a retrospective cohort study, included 147 participants operated on between 2002 and 2011. The aim was to evaluate the long-term clinical outcomes after arthroscopic Bankart repair, assess the rate of instability recurrence, and identify factors associated with surgical failure—among them, the number of anchors used. The mean follow-up was six years. Most patients were men, with a mean age of 26 years, and the most frequent cause of the lesions was trauma. The surgical technique consisted of mobilizing the capsulolabral complex, reinserting the labrum onto the anterior glenoid rim, and fixation with suture anchors, grouped as two anchors, three anchors, and four or more anchors. The primary outcome was instability recurrence, defined as a new dislocation or recurrent subluxation; secondary outcomes included the Western Ontario Shoulder Instability Index (WOSI), the Simple Shoulder Test, and the SF-12.

The authors observed an overall recurrence rate of 22% and reported that patients treated with fewer than three anchors had a higher recurrence rate. Despite this association, the study emphasized that the number of anchors is not the sole determinant of failure; other relevant factors included young age, contact sports, the number of previous dislocations, and possible glenoid bone loss.^11^ Özdemir et al.,^12^ in a retrospective cohort study, evaluated 40 patients (38 men and 2 women; mean age 31.6 ± 8.1 years; range 17–47) who underwent arthroscopic Bankart repair and were followed for a minimum of two years. Group 1 (n = 17) underwent repair with two suture anchors and Group 2 (n = 23) with three suture anchors. A significant improvement in functional outcomes was observed in all patients at final follow-up. No statistically significant difference (p > 0.05) was found between groups in the mean Constant Shoulder Score (Group 1: 94.2 ± 7.8; Group 2: 95.4 ± 4.1), the mean Rowe score (Group 1: 95.6 ± 4.6; Group 2: 96.3 ± 3.8), or the loss of external rotation in neutral position (Group 1: 1.9°; Group 2: 2.2°). Recurrent dislocation occurred in three patients (n = 2 in Group 1 and n = 1 in Group 2). The authors concluded that glenohumeral stability was not correlated with the use of two or three anchors in arthroscopic Bankart repair.

The general characteristics of the included studies and the certainty of evidence according to the Grading of Recommendations Assessment, Development and Evaluation (GRADE) approach are presented in Tables 1 and 2, respectively.^13,14^

**Table 1:** Characteristics of the included studies.

Included study	Study design	Sample size(n)	Follow-up
Buckup J. et al.^5^	Randomized controlled trial	40 (20 vs 20)	12 months (MRI); 24 months (clinical)
Vermeulen A.E. et al.^11^	Retrospective cohort	147	Mean 6 years
Özdemir B. et al.^12^	Retrospective cohort	40 (17 vs 23)	Minimum 2 years

MRI: magnetic resonance imaging.

**Table 2:** Quality of evidence (GRADE): three or more anchors versus up to two anchors.

Outcome	Studies (N)	Certainty of evidence
Recurrence of instability	3 studies (227)	Very low^1^,^2^,^3^
Function (functional scores)	3 studies (227)	Very low^1^,^2^,^3^
Complications	2 studies (80)	Very low^1^,^2^,^3^

1 Limited sample size;

2 Imprecision (wide confidence interval);

3 Risk of bias of the included studies (predominantly observational design). Note: GRADE (Grading of Recommendations Assessment, Development and Evaluation) is a tool used to assess and grade the certainty of evidence, based on five domains: risk of bias, imprecision, inconsistency, indirectness, and publication bias. The certainty of evidence is classified as very low, low, moderate, or high.

### Study Limitations

Future research should prioritize randomized, multicenter clinical trials with rigorous methodology. Studies with this design are essential to more precisely define the long-term clinical and functional outcomes associated with each technique, since no studies with high methodological rigor on this topic were identified.

### Recommendation

Regarding functional outcomes and complications, there is no evidence demonstrating that the use of three or more anchors is more effective than the use of two anchors for the arthroscopic treatment of anterior shoulder instability in adults. However, regarding the outcome of recurrence, there is evidence, albeit of very low quality, that the use of three or more anchors is more effective than the use of up to two anchors.

Therefore, the Brazilian Society of Shoulder and Elbow Surgery (SBCOC) Consensus Project recommends that the surgeon individually assess patient- and disease-related characteristics to reach the best treatment decision. With respect to recurrence, this project recommends that the surgeon give preference to the use of three or more anchors for the treatment of anterior shoulder instability, given the trend toward a lower recurrence rate.

## CONCLUSION

Regarding functional outcomes and complications, there is no evidence demonstrating that the use of three or more anchors is more effective than the use of two anchors for the arthroscopic treatment of anterior shoulder instability in adults. However, regarding recurrence, there is evidence, albeit of very low quality, that the use of three or more anchors is more effective than the use of up to two anchors.

## Data Availability

The contents underlying the research are available in the manuscript.

## Appendix 1 Database search strategy.

**Table:**

**PUBMED**
#1 "Shoulder Dislocation"[Mesh] OR (Dislocation, Shoulder) OR (Dislocations, Shoulder) OR (Shoulder Dislocations) OR (Glenohumeral Dislocation) OR (Dislocation, Glenohumeral) OR (Dislocations, Glenohumeral) OR (Glenohumeral Dislocations) OR (Glenohumeral Subluxation) OR (Glenohumeral Subluxations) OR (Subluxation, Glenohumeral) OR (Subluxations, Glenohumeral)
#2 "Joint Instability"[Mesh] OR (Instability, Joint) OR (Instabilities, Joint) OR (Joint Instabilities) OR (Hypermobility, Joint) OR (Hypermobilities, Joint) OR (Joint Hypermobilities) OR (Joint Hypermobility) OR (Laxity, Joint) OR (Joint Laxities) OR (Joint Laxity) OR (Laxities, Joint)
#3 "Arthroscopy"[Mesh] OR (Arthroscopies) OR (Arthroscopic Surgical Procedures) OR (Arthroscopic Surgical Procedure) OR (Procedure, Arthroscopic Surgical) OR (Procedures, Arthroscopic Surgical) OR (Surgical Procedure, Arthroscopic) OR (Arthroscopic Surgery) OR (Arthroscopic Surgeries) OR (Surgeries, Arthroscopic) OR (Surgery, Arthroscopic) OR (Surgical Procedures, Arthroscopic)
#4 "Tissue Fixation"[Mesh] OR (Fixation, Tissue)
#5 "Suture Anchors"[Mesh] OR (Anchors, Suture) OR (Anchor, Suture) OR (Suture Anchor) OR (Bone Anchors) OR (Anchor, Bone) OR (Anchors, Bone) OR (Bone Anchor)
#6 "Recovery of Function"[Mesh] OR (Function Recoveries) OR (Function Recovery) OR "Postoperative Complications"[Mesh] OR (Complication, Postoperative) OR (Complications, Postoperative) OR (Postoperative Complication) OR "Recurrence"[Mesh] OR (Recurrences) OR (Relapse) OR (Relapses) OR (Recrudescence) OR (Recrudescences)
#7 #1 OR #2
#8 #3 OR #4
#9 #7 AND #8 AND #5 AND #6 - 279

**Table:**

**COCHRANE LIBRARY**
#1 (Shoulder Dislocation) OR (Dislocation, Shoulder) OR (Dislocations, Shoulder) OR (Shoulder Dislocations) OR (Glenohumeral Dislocation) OR (Dislocation, Glenohumeral) OR (Dislocations, Glenohumeral) OR (Glenohumeral Dislocations) OR (Glenohumeral Subluxation) OR (Glenohumeral Subluxations) OR (Subluxation, Glenohumeral) OR (Subluxations, Glenohumeral)
#2 (Joint Instability) OR (Instability, Joint) OR (Instabilities, Joint) OR (Joint Instabilities) OR (Hypermobility, Joint) OR (Hypermobilities, Joint) OR (Joint Hypermobilities) OR (Joint Hypermobility) OR (Laxity, Joint) OR (Joint Laxities) OR (Joint Laxity) OR (Laxities, Joint)
#3 (Arthroscopy) OR (Arthroscopies) OR (Arthroscopic Surgical Procedures) OR (Arthroscopic Surgical Procedure) OR (Procedure, Arthroscopic Surgical) OR (Procedures, Arthroscopic Surgical) OR (Surgical Procedure, Arthroscopic) OR (Arthroscopic Surgery) OR (Arthroscopic Surgeries) OR (Surgeries, Arthroscopic) OR (Surgery, Arthroscopic) OR (Surgical Procedures, Arthroscopic)
#4 (Tissue Fixation) OR (Fixation, Tissue)
#5 (Suture Anchors) OR (Anchors, Suture) OR (Anchor, Suture) OR (Suture Anchor) OR (Bone Anchors) OR (Anchor, Bone) OR (Anchors, Bone) OR (Bone Anchor)
#6 #1 OR #2
#7 #3 OR #4
#8 #6 AND #7 AND #5

**Table:**

**EMBASE**
#1 ‘shoulder dislocation’/exp OR ‘dislocation, shoulder’ OR ‘humeroscapular luxation’ OR ‘scapulohumeral luxation’ OR ‘shoulder dislocation’ OR ‘shoulder joint dislocation’ OR ‘shoulder luxation’
#2 ‘joint instability’/exp OR ‘instability, joint’ OR ‘joint instability’
#3 ‘arthroscopy’/exp OR ‘arthro-endoscopy’ OR ‘arthroendoscopy’ OR ‘arthroscopic exam’ OR ‘arthroscopic examination’ OR ‘arthroscopic procedure’ OR ‘arthroscopic procedures’ OR ‘arthroscopy’
#4 ‘tissue fixation’/exp OR ‘tissue fixation’
#5 ‘suture anchor’/exp OR ‘bioraptor’ OR ‘betta link’ OR ‘bio-corkscrew’ OR ‘bio-suture tak’ OR ‘bio-suturetak’ OR ‘biocorkscrew’ OR ‘biowick’ OR ‘bioplug nt’ OR ‘biosteon intraline’ OR ‘chait (suture anchor)’ OR ‘compositcp (suture anchor)’ OR ‘cope (suture anchor)’ OR ‘corkscrew (suture anchor)’ OR ‘crossft bc’ OR ‘fastak’ OR ‘fiberstitch’ OR ‘fibertak’ OR ‘fibulink’ OR ‘footprint pk’ OR ‘gii’ OR ‘graftrope’ OR ‘gravity (suture anchor)’ OR ‘gryphon’ OR ‘gryphon proknot’ OR ‘healicoil (suture anchor)’ OR ‘healicoil pk’ OR ‘healicoil’ OR ‘healix (suture anchor)’ OR ‘healix advance’ OR ‘healix ti’ OR ‘healix transtend’ OR ‘iconix’ OR ‘iconix speed’ OR ‘insite ft’ OR ‘juggerknot’ OR ‘juggerknotless’ OR ‘juggerstitch’ OR ‘knotilus’ OR ‘lupine (suture anchor)’ OR ‘memofix’ OR ‘memosorb’ OR ‘micro quickanchor’ OR ‘microfix’ OR ‘mini quickanchor’ OR ‘minilok’ OR ‘morphix’ OR ‘nanotack tt’ OR ‘omega (suture anchor)’ OR ‘omnispan’ OR ‘peek intraline’ OR ‘peek zip’ OR ‘piton (suture anchor)’ OR ‘poplok’ OR ‘pressft’ OR ‘procinch’ OR ‘pushlock’ OR ‘q-fix’ OR ‘quattro (suture anchor)’ OR ‘quattro gl’ OR ‘rigidfix’ OR ‘reelx’ OR ‘reelx stt’ OR ‘retrobutton’ OR ‘rigidloop’ OR ‘speedlock (suture anchor)’ OR ‘speedscrew’ OR ‘suturefix ultra’ OR ‘suturefix ultra xl’ OR ‘sonicanchor’ OR ‘sonicpin’ OR ‘surelock’ OR ‘suturetak’ OR ‘swivelock’ OR ‘swivelock c’ OR ‘swivelock sp’ OR ‘swivelock tenodesis’ OR ‘twinfix ultra ha’ OR ‘twinfix ultra pk’ OR ‘tacit’ OR ‘tensionloc’ OR ‘truespan’ OR ‘twinfix’ OR ‘twinloop flex’ OR ‘ventix’ OR ‘versalok’ OR ‘versaloop’ OR ‘y-knot’ OR ‘ziptight’ OR ‘bioabsorbable soft tissue anchor’ OR ‘bioabsorbable soft-tissue anchor’ OR ‘bioabsorbable suture anchor’ OR ‘bone anchor’ OR ‘bone anchors’ OR ‘ibalance’ OR ‘non-bioabsorbable soft-tissue anchor’ OR ‘soft-tissue anchor, bioabsorbable’ OR ‘soft-tissue anchor, non-bioabsorbable’ OR ‘surgical binding material anchor’ OR ‘surgical binding material anchor set’ OR ‘suture anchor’ OR ‘suture anchors’
#6 ‘orthopedic fixation device’/exp OR ‘acumed’ OR ‘biofoam (orthopedic fixation device)’ OR ‘tunneloc’ OR ‘veptr’ OR ‘veptr ii’ OR ‘fixation device’ OR ‘fixation device (attribute)’ OR ‘fixation devices’ OR ‘fixator’ OR ‘fixator (physical object)’ OR ‘fixators’ OR ‘immobiliser (orthopaedic)’ OR ‘immobilizer (orthopedic)’ OR ‘metallic osteotomy fusion cage’ OR ‘orthopaedic fixation device’ OR ‘orthopaedic fixation devices’ OR ‘orthopaedic fixation systems’ OR ‘orthopaedic immobiliser’ OR ‘orthopedic fixation device’ OR ‘orthopedic fixation devices’ OR ‘orthopedic immobilizer’ OR ‘surgical fixation devices’
#7 #1 OR #2
#8 #3 OR #4
#9 #5 OR #6
#10 #7 AND #8 AND #9 - 1875
#11 AND [embase]/lim NOT ([embase]/lim AND [medline]/lim) - 791
#12 ‘convalescence’/exp OR ‘convalescence’ OR ‘convalescence cure’ OR ‘convalescent’ OR ‘cure, convalescence’ OR ‘recovery of function’ OR ‘postoperative complication’/exp OR ‘complication after operation’ OR ‘complication after surgery’ OR ‘complication, postoperative’ OR ‘complication, surgical’ OR ‘post-operation complication’ OR ‘post-operative complication’ OR ‘post-operative complications’ OR ‘post-surgery complication’ OR ‘post-surgical complication’ OR ‘postoperation complication’ OR ‘postoperative complication’ OR ‘postoperative complications’ OR ‘postsurgery complication’ OR ‘postsurgical complication’ OR ‘surgery complication’ OR ‘surgery complications’ OR ‘surgery-associated complication’ OR ‘surgery-derived complication’ OR ‘surgery-induced complication’ OR ‘surgery-related complication’ OR ‘surgical complication’ OR ‘recurrent disease’/exp OR ‘disease recurrence’ OR ‘periodic disease’ OR ‘recurrence’ OR ‘recurrent disease’ OR ‘relapsing disease’ OR ‘symptom flare up’
#13 #11 AND #12

**Table:**

**REGIONAL PORTAL (BVS)**
#1 MH:"Luxação do Ombro" OR (Shoulder Dislocation) OR (Luxação Glenoumeral) OR MH:C05.550.518.750$ OR MH:C26.289.750$ OR MH:C26.803.125$ OR MH:"Instabilidade Articular" OR (Joint Instability) OR (Frouxidão Articular) OR (Frouxidão Ligamentar) OR (Frouxidão da Articulação) OR (Hiperfrouxidão Ligamentar Difusa) OR (Hipermobilidade Articular) OR (Hipermobilidade da Articulação) OR (Instabilidade da Articulação) OR MH:C05.550.521$
#2 MH:"Artroscopia" OR (Arthroscopy) OR (Procedimentos Cirúrgicos Artroscópicos) OR MH:E01.370.388.250.070$ OR MH:E04.502.250.070$ OR MH:E04.555.113$ OR MH:"Fixação de Tecidos" OR (Tissue Fixation) OR MH:E01.370.225.500.620.760.720$ OR MH:E01.370.225.750.600.760.720$ OR MH:E05.200.500.620.760.720$ OR MH:E05.200.750.600.760.720$
#3 MH:"Âncoras de Sutura" OR (Suture Anchors) OR (Ancoragem Óssea) OR (Âncoras Ósseas) OR MH:E07.695.370.734$ OR MH:E07.858.442.660.460.734$ OR MH:E07.858.690.725.460.734$
#4 #1 AND #2 AND #3
